# Penetration of sweet cherry skin by ^45^Ca-salts: pathways and factors

**DOI:** 10.1038/s41598-021-90727-0

**Published:** 2021-05-27

**Authors:** Andreas Winkler, Moritz Knoche

**Affiliations:** Institute of Horticultural Production Systems, Leibniz University Hanover, Herrenhäuser Straße 2, 30419 Hannover, Germany

**Keywords:** Biological techniques, Plant sciences

## Abstract

Calcium is beneficial to sweet cherry physiology. The objective was to investigate factors affecting uptake of Ca into mature sweet cherry fruit through their skins. Penetration of ^45^Ca-salts was monitored using whole fruit or excised fruit skins mounted in diffusion cells. Penetration of ^45^CaCl_2_ into intact fruit and through excised skins increased with time. Sealing the pedicel/fruit junction decreased penetration, but sealing the stylar scar had no effect. There was little difference in permeances of the fruit skin to ^45^CaCl_2_, ^45^Ca(NO_3_)_2_, ^45^Ca-formate, ^45^Ca-acetate, ^45^Ca-lactate or ^45^Ca-propionate. Only ^45^Ca-heptagluconate penetrated at a slower rate. Increasing temperature markedly increased Ca-penetration. Penetration was most rapid at 35 °C, intermediate at 22 °C and slowest at 12 °C. Increasing relative humidity (RH) from 0, 28, 75 to 100% increased penetration of ^45^CaCl_2_, but penetration of ^45^Ca-formate was restricted to 100% RH. Increasing the RH from 50 to 100% at 96 h after droplet application had no effect on penetration of ^45^CaCl_2_, but increased penetration of ^45^Ca-formate. The results reveal that: (1) the fruit/pedicel junction is a site of preferential Ca-uptake and (2) Ca-penetration is limited by the mobility of the Ca ion in the dried-down droplet residue when the point of deliquescence of the applied salt exceeds the ambient RH.

## Introduction

Calcium plays an important role in the pre- and postharvest physiology of sweet cherry fruit. High fruit Ca-content is considered beneficial for many properties, including firmness, susceptibility to rot and fruit cracking^1^. Calcium is transported only in the xylem^2^. In cherry, fruit xylem conductance falls to almost zero during development^3,4^. Hence, later fruit growth is driven exclusively by inflows of phloem sap. As a consequence, the concentration of Ca in the fruit decreases in the later stages of fruit development^5,6^. Sweet cherry is not unique in this respect^7–11^.

Spray applications of Ca-salts can be effective in increasing fruit Ca-content^12^. However, reports on the effectiveness of spray applications of Ca-salts in sweet cherry are inconsistent. In a number of studies, there was no effect on fruit physiology^13–15^. In others, Ca-sprays were even ineffective in increasing fruit Ca-content^13,16,17^. The lack of a significant Ca response may be due to low Ca uptake, for example as a result of adverse environmental conditions at the time of application—e.g. low temperatures, low RHs. Also, high natural fruit-to-fruit variability in Ca-content may mask the effects of Ca-sprays on fruit Ca-content. Fruit of the same batch (cultivar, site, sampling date) can differ two-fold in Ca-content^18^. Thus, small increases in Ca-content following spray application may not be detectable above this statistical noise. Large sample sizes and good randomization are required to detect minor changes in Ca with such high background variability. This makes such Ca-analyses both time-consuming and expensive.

Laboratory experiments using radioactive ^45^Ca as a tracer are an alternative way to quantify Ca-penetration. Because background levels of ^45^Ca in sweet cherry fruit and in (deionized) water are essentially zero, even very low levels of ^45^Ca penetration can be detected. Two different systems are described in the literature to quantify penetration under controlled conditions^19,20^.

First, penetration may be monitored from a dilute donor solution containing a ^45^Ca-salt through an interface of excised skin (ES) into a receiver solution. Because the concentration gradient across the ES remains essentially constant during the duration of an experiment, the system is referred to as the infinite-dose system^19^. Penetration is monitored by repeated sampling of the receiver solution. The infinite-dose system is particularly suited to quantifying and comparing permeances under steady-state conditions. Second, with finite-dose diffusion, penetration from a simulated spray droplet into a receiver solution is monitored through excised fruit skins. Here, as in the real world, a spray droplet is subject to drying out so a dry deposit is ultimately formed on the skin. Because the initial volume of the droplet is small relative to the receiver solution, this system is referred to as the finite-dose system^20^. As in the infinite-dose experiments, penetration is monitored by sampling the receiver solution. Unlike infinite-dose diffusion experiments, the concentration of the donor droplet and the cross-sectional area of the droplet and the deposit change continuously as penetration proceeds. Steady state conditions are usually not achieved. The system is used primarily to study effects on skin penetration of environment or of spray application factors^21^. In addition, whole fruit may be incubated in a dilute donor solution. Penetration is monitored by destructive sampling. Technically, this system represents an infinite-dose system, because the concentration in the donor solution remains essentially constant during penetration. When operated over short periods, the tissue concentration of ^45^Ca also remains low, so the concentration gradient is essentially constant. Because the fruit remains intact, this system is useful for identifying sites of preferential penetration.

The objectives of this study were: (1) to identify any sites of preferential Ca-uptake into intact sweet cherry fruit, (2) to investigate the effects of Ca-salts having different anions on penetration through excised fruit skins, and (3) to quantify the effects of temperature, RH and the re-wetting of a dried droplet deposit on penetration of ^45^Ca. For objectives (2) and (3) we used infinite- and finite-dose diffusion cells, where penetration of ^45^Ca was monitored through excised ES under controlled conditions.

## Results

Sealing the pedicel cavity decreased the penetration of ^45^Ca into sweet cherry fruit, whereas sealing the stylar scar had no effect. Here, the effect was significant for ‘Burlat’, but not for ‘Regina’ (Table 1).

**Table 1:** Effects of selective sealing of potential sites of preferential uptake on penetration of ^45^CaCl_2_ into whole sweet cherry fruit.

Region sealed	Penetration (Bq)		
	Burlat	Regina	Mean
Control	172 ± 24 a^z^	132 ± 17 a	153 ± 15 b
Pedicel cavity	57 ± 4 b	97 ± 9 a	77 ± 6 a
Stylar scar	141 ± 14 a	152 ± 22 a	146 ± 13 b

Fruit was incubated for 4 h in 50 mM CaCl_2_ at a radioactivity concentration of 3.3 kBq ml^−1^. The pedicel cavity or the stylar scar region were sealed with a fast-curing silicone rubber.

^z^Mean separation within columns by Tukey’s Studentised range test at p ≤ 0.05.

**Figure 1:**
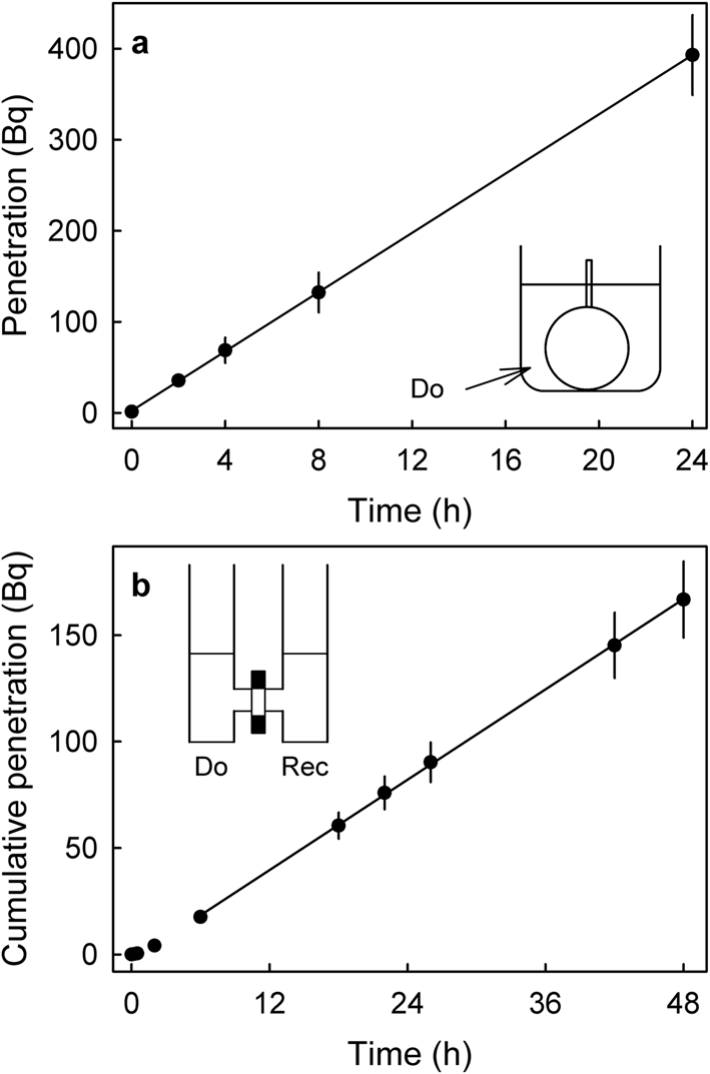
Time courses of penetration of ^45^CaCl_2_ in sweet cherry. (**a**) Whole fruit incubated in a ^45^CaCl_2_ containing donor solution. Inset: Sketch of a sweet cherry fruit incubated in ^45^CaCl_2_. (**b**) Cumulative penetration from a donor (Do) solution containing ^45^CaCl_2_ through an excised skin segment into a receiver (Rec) solution. Inset: Sketch of an infinite diffusion cell.

**Figure 2:**
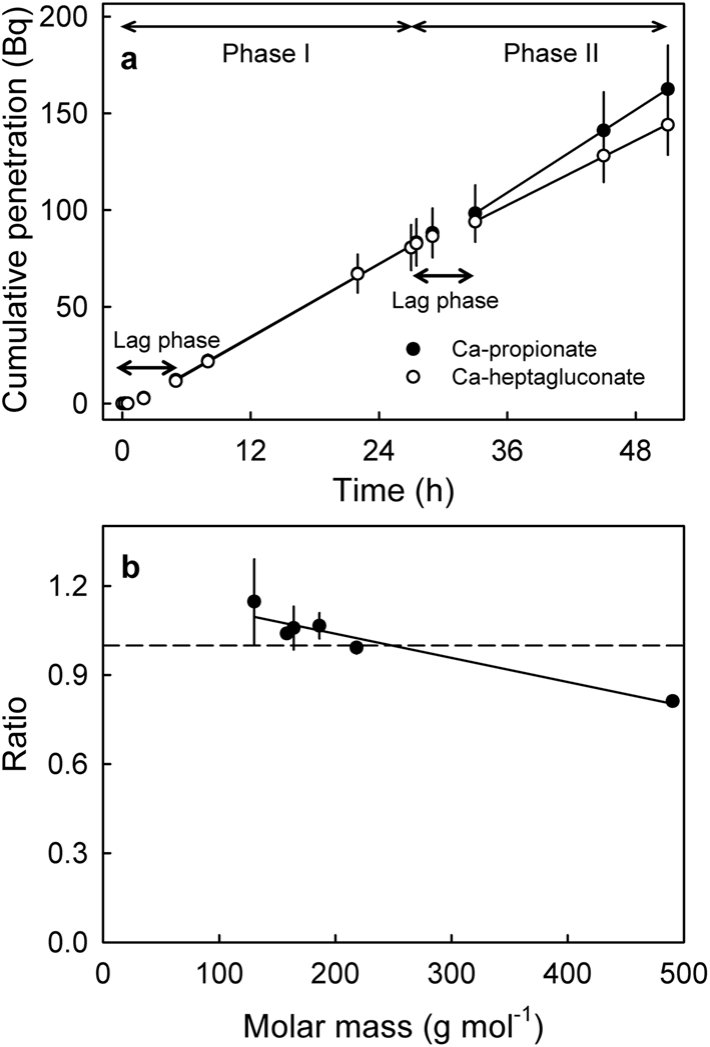
(**a**) Time course of penetration of ^45^Ca-salts from a donor solution through an excised skin segment of mature sweet cherry fruit into a receiver solution. In phase I of the experiment, the steady state flow rate of ^45^Ca was established using ^45^CaCl_2_ as the donor solution. At the beginning of phase II, the ^45^CaCl_2_ donor solution was replaced by one of the following salt solutions: ^45^Ca(NO_3_)_2_, ^45^Ca-formate, ^45^Ca-acetate, ^45^Ca-lactate, ^45^Ca-heptagluconate or ^45^Ca-propionate. The steady state flow rates were re-established. Steady states flow rates and permeances were calculated from the slopes of regression lines fitted through the linear portions of plots of cumulative penetration vs. time. From these flow rates, permeances were calculated (for details see “Material and methods”). For permeance estimates see Table 2. (**b**) Ratio of permeances during phase II divided by phase I (^45^CaCl_2_) as a function of molecular size. The dashed line represents the ratio value 1.0, where the permeance during phase II is identical to that during phase I.

The time course of ^45^CaCl_2_ penetration revealed linear increases in penetration for up to 24 h into intact sweet cherry fruit and for up to 48 h in infinite-dose penetration through an excised sweet cherry fruit skin, indicating that the rates of penetration remained constant (Fig. 1a,b). Replacing the ^45^CaCl_2_ by other Ca-salts had little effect on penetration. Only the heptagluconate anion significantly decreased the rate of penetration (Fig. 2a, Table 2). There was a slight trend for decreasing penetration of ^45^Ca as the molar mass of the anion increased (Fig. 2b).

**Table 2:** Permeance estimates of different Ca-salts through excised skin segments of mature sweet cherry fruit.

Donor	Permeance (× 10^–9^ m s^−1^)		Ratio
Phase I/phase II	Phase I	Phase II	
^45^CaCl_2_/^45^Ca-acetate	1.13 ± 0.17	1.17 ± 0.18	1.04 ± 0.02
^45^CaCl_2_/^45^Ca-formate	1.86 ± 0.34	2.08 ± 0.44	1.15 ± 0.14
^45^CaCl_2_/^45^Ca-heptagluconate	1.70 ± 0.19	1.37 ± 0.14*	0.81 ± 0.01
^45^CaCl_2_/^45^Ca-lactate	1.17 ± 0.12	1.15 ± 0.12	0.99 ± 0.02
^45^CaCl_2_/^45^Ca(NO_3_)_2_	1.68 ± 0.21	1.77 ± 0.24	1.06 ± 0.07
^45^CaCl_2_/^45^Ca-propionate	1.69 ± 0.25	1.75 ± 0.22	1.07 ± 0.04

During phase I of the experiment, ^45^CaCl_2_ was used as the donor solution. In phase II, the donor solution was replaced by ^45^Ca(NO_3_)_2_ or by one of the organic salts ^45^Ca-acetate, ^45^Ca-formate, ^45^Ca-heptagluconate, ^45^Ca-lactate or ^45^Ca-propionate. The change in permeance between phases I and II is specified as the ratio phase II/phase I (for an example of the time course see Fig. 2).

*Significantly different from the permeance in phase I. The other phase I: phase II pairs are not significantly different. Paired Student’s t test at p ≤ 0.05.

**Figure 3:**
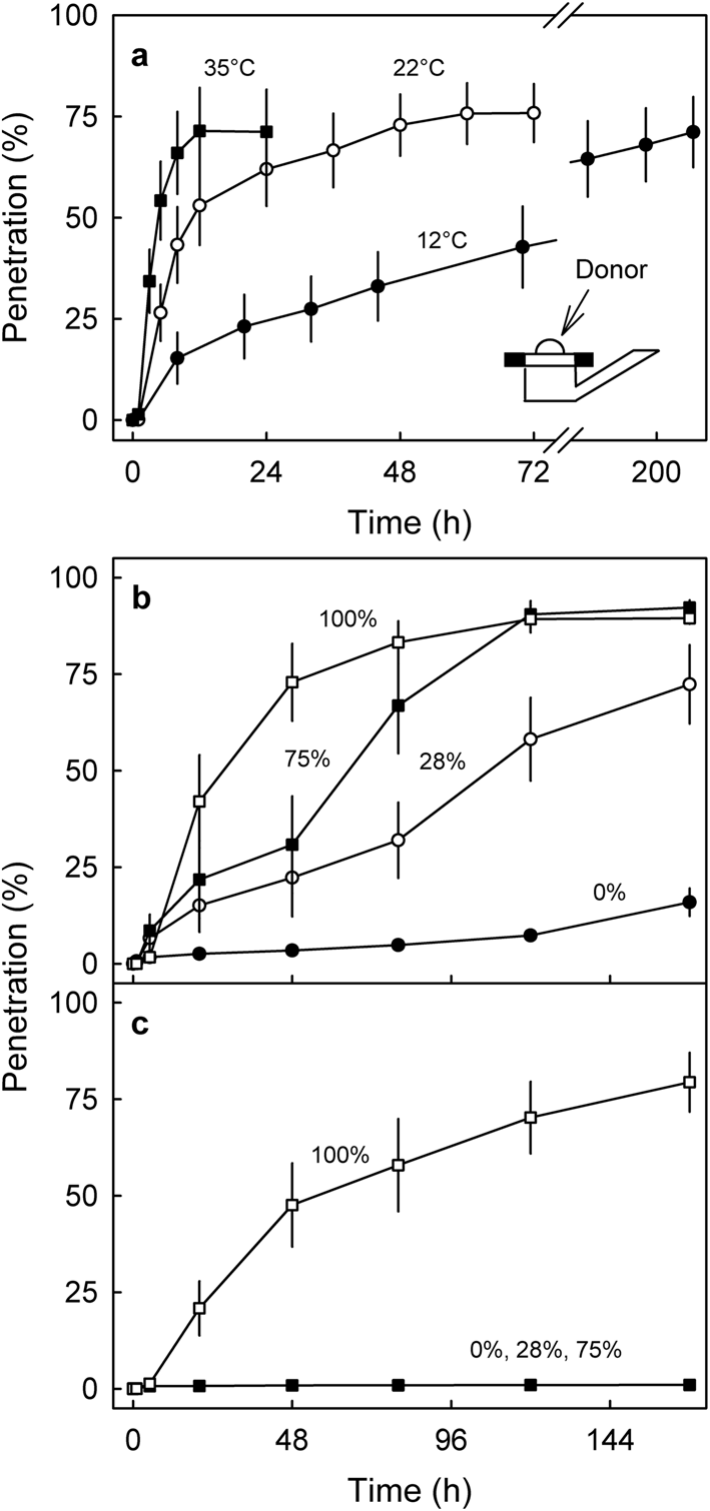
(**a**) Effect of temperature on the time course of penetration of ^45^CaCl_2_ through excised skin segments of mature sweet cherry fruit. Relative humidity (RH) was maintained constant at 75%. Inset: Sketch of a finite-dose diffusion cell. (**b**,**c**) Effect of RH on the time course of penetration of ^45^CaCl_2_ (**b**) or ^45^Ca-formate (**c**). Temperature was maintained constant at 22 °C and RH was adjusted by holding diffusion cells above dry silica gel (0% RH), saturated CaCl_2_ (28% RH), saturated NaCl (75% RH) or water (100% RH). Please note, that the data symbols for 0, 28 and 75% RH are all superimposed in (**c**).

Temperature had a marked effect on penetration of ^45^CaCl_2_ (Fig. 3a). Penetration was most rapid at 35 °C, intermediate at 22 °C and least at 12 °C. Half maximum penetration (50% of the amount applied) was reached after 4.6 h at 35 °C, after 10.9 h at 22 °C and after 96 h at 12 °C (Fig. 3a).

The RH had a marked effect on penetration of ^45^CaCl_2_ and ^45^Ca-formate. The rate of penetration of ^45^CaCl_2_ increased with increasing RH (Fig. 3b). In contrast, penetration of ^45^Ca-formate occurred only at an RH of 100% RH, there was essentially no penetration at 0, 28 or 75% RH (Fig. 3c).

**Figure 4:**
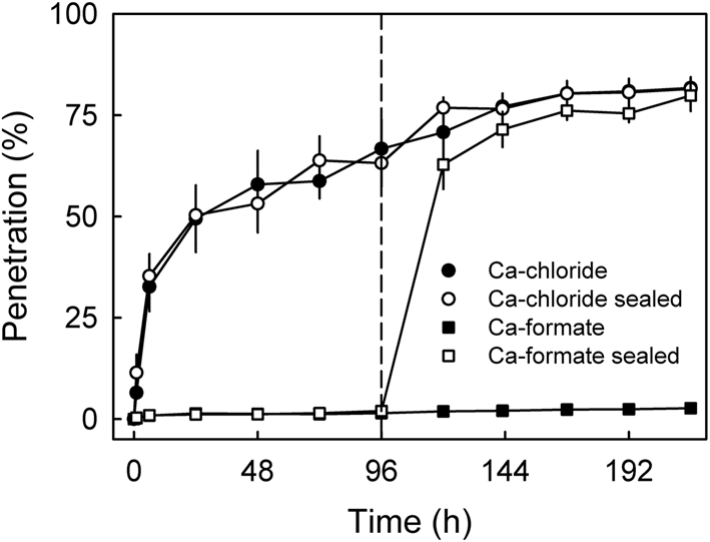
The effect of increasing the relative humidity (RH) (from 50 to 100%) on penetration of ^45^CaCl_2_ and ^45^Ca-formate through excised skin segments of mature sweet cherry fruit at 96 h after droplet application. Penetration was monitored at 22 °C and 50% RH.

Increasing the RH from 50 to 100%, 96 h after droplet application, had no effect on penetration of ^45^CaCl_2_ compared to the control that remained at 50% RH. However, when carrying out the same experiment using ^45^Ca-formate, increasing the RH from 50 to 100% markedly increased penetration. After 120 h, penetration of ^45^Ca-formate at 100% RH had reached about 60%. At 216 h an asymptote was reached where penetration of ^45^Ca-formate at 100% did not differ from penetration of ^45^CaCl_2_. In contrast, penetration at 50% RH remained negligibly low (Fig. 4).

**Figure 5:**
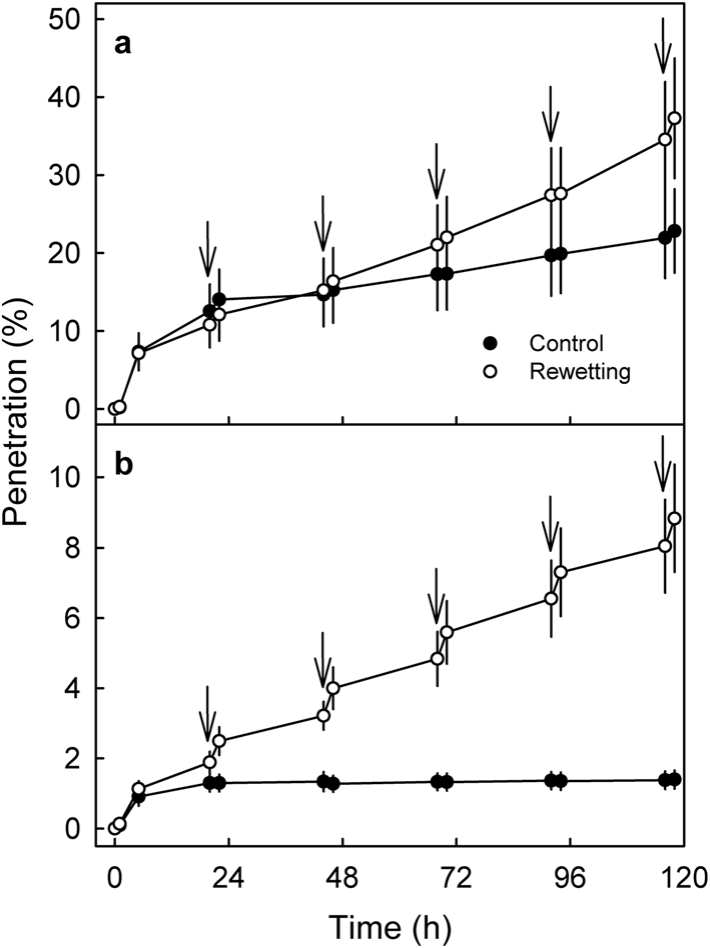
Effect of re-wetting the dried-down droplet deposit on penetration of ^45^CaCl_2_ (**a**) and ^45^Ca-formate (**b**) through excised skin segments of mature sweet cherry fruit. The droplet deposit was re-wetted daily (indicated by arrows) and penetration was monitored at 22 °C and 50% relative humidity.

Re-wetting the dried-down droplet residue had little effect on penetration of ^45^CaCl_2_ (Fig. 5a), but markedly increased penetration of ^45^Ca-formate. Penetration of ^45^Ca-formate quickly stopped when the droplet had dried (Fig. 5b).

## Discussion

The model systems employed in this study proved to be useful tools for quantifying penetration of ^45^Ca under controlled conditions. Using these systems, we demonstrate that (1) the pedicel cavity, but not the stylar scar region, is a site of preferential Ca-uptake, (2) there is generally little difference in penetration between the different Ca-salt anions and (3) penetration of Ca from a drying spray droplet is limited by the mobility of Ca in the dried droplet deposit.

Using whole fruit, the pedicel cavity was identified as a site of preferential penetration. By using radiolabeled Ca, the penetration of ^45^Ca is accurately measurable, even if the fruit content of the common isotope ^40^Ca is highly variable (as it is in sweet cherry^6^). By ashing the fruit, we minimized the handling of label. The whole-fruit system had the advantage of being readily available and not requiring any manipulations. In addition, regions of the fruit surface that are not suitable for preparing skin segments, such as the pedicel cavity with the pedicel/fruit junction, can be investigated. However, a disadvantage is that the system requires destructive sampling. This eliminates the possibility of repeat observations on individual fruits. Our experiments demonstrate that Ca-penetration occurred to a significant extent in the pedicel cavity region. It is interesting, that the same observation has been made for water uptake into sweet cherry fruit^22^. The cuticle in the pedicel cavity is discontinuous at the junction between the fruit surface and the pedicel. Water and solutes, including Ca ions, can penetrate freely along the gap at the pedicel/fruit junction. As in water uptake, Ca-penetration along this pathway is highly variable as indicated by the consistently large standard errors on all treatments involving junction penetration. The amount of water penetrating along the junction increases during fruit maturation and ripening and is negatively related to the fruit removal force^22^. We would expect the same to apply for Ca uptake.

The steady-state conditions in the infinite-dose system allows experimental designs involving repeated measurements to be made on each ES, including sequential measurements of control and treatment. This enabled critical assessments of permeance changes when the CaCl_2_ containing donor solution of the control was replaced by one containing a different Ca-salt. With a single exception, we observed little difference in permeance between the Ca salts tested. While ^45^Ca was the cation monitored for penetration, any ^45^Ca gaining entry must have been accompanied by an anion partner to maintain electrical neutrality^23^. In general, both cations and anions are excluded from the lipophilic pathway across the cuticle because of their charge. Their passage is normally restricted to the polar pathway, which is also present in sweet cherry fruit^24^. Due to the porous nature of the polar pathway, we would expect penetration of Ca-salts to decrease to zero as molar mass exceeds the size exclusion limit of the polar pathway. Our data indicate the size exclusion limit is approached, but not exceeded, by the largest Ca-salt tested here (Ca-heptagluconate, anhydrous molar mass 492 g mol^−1^). Compared to CaCl_2_, Ca-heptagluconate penetrated at a significantly reduced rate—but only slightly lower. A size conclusion limit > 492 g mol^−1^ is consistent with an earlier study, where the size exclusion limit in sweet cherry was found to lie somewhere between sucrose (the largest penetrating solute, 342 g mol^−1^) and polyethylene glycol 1500 (the smallest non-penetrating solute, 1500 g mol^−1^)^24^. It is important to note that polar pathways do not represent perfect cylindrical holes of constant diameter. More likely, they follow a size distribution that is likely to be also affected by cuticle hydration.

The finite-dose system allows us to study the effects of re-wetting of the deposit and of environmental variables such as temperature and RH. Since the simulated spray droplet was subject to drying, we investigated penetration from both, the liquid phase and the dry-deposit phase. The results demonstrate that Ca penetration is limited by its mobility in the dried deposit. By increasing the RH beyond the point of deliquescence (DQ) of the salt, Ca penetration was increased^23,25^. When the RH is above the DQ, the droplet remains as a concentrated solution. This explains why, for Ca-formate (DQ > 95% RH^26^), there was no effect of RH on penetration between RH 0 and 75%. Only at RH 100% was penetration markedly increased. In contrast, for CaCl_2_ (DQ RH 28%^27^) penetration increased as RH increased from 0 to 100%. The marked effect of temperature on penetration is somewhat surprising. We would have expected penetration along the polar pathways to be largely independent of temperature. Penetration along the polar pathway is primarily by viscous flow, where temperature effects are limited to small changes in the viscosity of water^28^. This view is also consistent with the low energy of activation for water penetration across the sweet cherry fruit skin reported previously^24^. A possible explanation for the effect of temperature is an indirect effect on cuticle and/or deposit hydration^25,29^. The temperature experiment was carried out at a constant RH of 75%. Under these conditions, the water vapor concentration (the absolute humidity) driving hydration is highest at the highest temperature. For constant RH, the absolute humidity decreases as temperature decreases. Hence, effects of temperature and cuticle and deposit hydration may have been confounded.

## Conclusion

From a practical point of view, maximum Ca-penetration may be achieved with any of the Ca-salts investigated here. The only exception was Ca-heptagluconate, where penetration was decreased. But, unless the DQ of the salt is low, as is the case of CaCl_2_, a surfactant must be added that maintains Ca mobility in the deposit. Ideally, penetration of the surfactant matches that of the Ca salt. This will maintain a high driving force for penetration of the Ca salt from the deposit. In the absence of a surfactant, penetration will likely be limited to humidities above the DQ of the respective salt. We expect the relationships identified herein to also apply to earlier stages of development. A previous study demonstrated that Ca penetration slighltly increases with development as surface area increases^6^.

At present, we do not know whether physiological effects of Ca applications are simply proportional to the amount of Ca taken up or whether the salt and hence, the anion associated with the Ca, also affects the physiology, e.g., fruit cracking and fruit firmness. To our knowledge, these aspects have not been fully addressed and hence, deserve closer attention.

## Material and methods

### Plant material

Sweet cherry fruit of the cultivars Burlat, Regina and Sweetheart were sampled at commercial maturity based on color and size from greenhouse-grown or field-grown trees grafted on ‘Gisela 5’ rootstocks (*P. cerasus* L. × *P. canescens* Bois) at the Horticultural Research Station of Leibniz University in Ruthe, Germany (lat. 52°14′N, long. 9°49′E). Fruit was processed on the day of sampling.

### Whole-fruit uptake

Fruit were incubated in a 50 mM CaCl_2_ donor solution that was spiked with the chloride of the radioactive isotope ^45^Ca (specific activity 30.5 GBq mmol^−1^; PerkinElmer, Waltham, MA, USA). The concentrations of radioactivity ranged from 3.3 to 3.6 KBq ml^−1^. The fruit was incubated such that the cut proximal end of the pedicel extended above the surface of the incubation solution. This prevented any uptake through the cut pedicel surface. Fruit were incubated for various periods before destructive sampling. For sampling, fruit were dipped three-times (each for 10 s) in deionized water to remove any radiolabel adhering to the fruit surface. Cracked fruit were discarded. Thereafter, fruit were carefully blotted, dried over silica gel, then ashed at 500 °C (ramped from 22 to 500 °C over 2 h, then held at 500 °C for 4 h) in glass scintillation vials in a muffle furnace (L24/11/B180; Nabertherm, Lilienthal, Germany). Preliminary experiments established that ashing temperatures and durations were sufficient to produce a whitish ash residue. Only occasionally was the residue black, indicating incomplete combustion. In these cases, the residue was taken up in 0.5 ml of 1 N HCl and re-ashed using the same settings. The ash was then taken up in 1 ml of 1 N HCl. Following the addition of scintillation liquid (scintillation cocktail Ultima Gold XR; PerkinElmer), the radioactivity was determined in a liquid scintillation spectrometer (LS 6500; Beckman Instruments, Fullerton, CA, USA). Using this procedure, the following experiments were carried out.

A time course of penetration of ^45^CaCl_2_ was established using ‘Regina’ by incubating fruit for 2, 4, 8 or 24 h. The number of replications was 12–15.

Sites of preferential uptake of ^45^CaCl_2_ were identified by selective sealing using a fast-curing silicone rubber (Dow Corning SE 9186; Dow Corning Corp., Midland, MI, USA) in ‘Burlat’ and ‘Regina’. The following regions were sealed: (1) pedicel cavity and pedicel and (2) stylar scar. Unsealed fruit served as controls. The number of replications was 11–15.

### Infinite-dose experiments

Infinite-dose diffusion cells were used to quantify penetration of different Ca-salts from a dilute donor solution through an interfacing ES into a receiver solution^20,24,30^. Briefly, an ES comprising cuticle, epidermis, hypodermis and several layers of subtending parenchyma was obtained from a mature sweet cherry fruit using a biopsy punch (10 mm diameter). The ES were hand cut to about 1 mm thickness using a razor blade and blotted using tissue paper. Subsequently, the ES were mounted in a polymethylmethacrylate (PMMA) holder using high-vacuum grease (Korasilon Paste hochviskos; Kurt Obermeier, Bad Berleburg, Germany). The cross-sectional area of the ES exposed in the holder was 19.6 mm^2^. The holders were positioned between two glass half-cells of an infinite-dose diffusion cell, such that the cuticle side faced the donor cell. The cells were placed on a multiple stirring plate and a stirring bar added to each half-cell. A leak-check was made by applying a slight hydrostatic pressure across the ES using 50 mM CaCl_2_. The ES of cells that did not maintain the gradient in hydrostatic pressure were replaced. The diffusion experiment was started by adding 5 ml of 50 mM CaCl_2_ as a receiver and 50 mM CaCl_2_ containing ^45^CaCl_2_ at a radioactivity concentration of 26.7 KBq ml^−1^ as the donor. Aliquots of 1 ml were removed from the receiver cell at regular intervals, radio-assayed by liquid scintillation spectrometry (scintillation cocktail Ultima Gold XR; PerkinElmer; counter: LS 6500; Beckman Instruments) and replaced by cold receiver solution.

The effect of the different Ca-salt anions on penetration of ^45^Ca was investigated in a two-phase experiment using a repeated-measures design and ‘Regina’ fruit. During phase I of the experiment, steady-state flow rates of ^45^CaCl_2_ were established as described above. Thereafter, phase II was initiated by replacing the CaCl_2_ donor by a donor solution containing either one of the following salts: Ca(NO_3_)_2_, Ca-formate, Ca-acetate, Ca-lactate, Ca-heptagluconate or Ca-propionate. The receiver was replaced by the same Ca-solution containing no ^45^Ca. The radioactive concentration was maintained constant. Steady state flow rates were re-established for phase II. The steady state flow rate ($F$, Bq s^−1^) was obtained by fitting a linear regression through a plot of cumulative ^45^Ca-penetration vs. time. The permeance ($P$, m s^−1^) of the skin was calculated using Fick’s law.

$$P=\frac{F}{A*\mathrm{\Delta C}}$$

In this equation, $A$ (m^2^) represents the cross-sectional area of the ES exposed in the PMMA holder and $\Delta C$ (Bq m^−3^) the difference in the concentrations of radioactivity between donor and receiver solutions. Because the amount of radioactivity penetrating from the donor into the receiver solution was very low, ΔC remained essentially constant and equal to the concentration of radioactivity in the donor solution. The number of replicates was eight.

### Finite-dose experiments

Finite-dose experiments were carried out as described before^20^. Epidermal skin segments were excised using a cork borer (15 mm inner diameter), hand cut to 1 mm thickness using a razor blade and blotted using tissue paper. The ES were mounted in PMMA holders using silicone grease (Korasilon Paste hochviskos; Kurt Obermeier). The holders were affixed to the top of a glass finite-dose diffusion cell^20^. A stirring bar was added and the holders with the ES positioned on the diffusion cell using silicone grease (Korasilon Paste hochviskos; Kurt Obermeier). About 3 ml of receiver solution (50 mM CaCl_2_ or Ca-formate) was added through the sampling port and the cells were placed on a multi-stirring plate. Following equilibration for a minimum of 1 h, a 5 µl droplet of a 50 mM CaCl_2_ or Ca-formate donor solution was applied to the outer side of the ES. The respective donor solutions were spiked with ^45^Ca so that the concentration of radioactivity was 16.7 KBq per 5 µl droplet. The receiver was sampled regularly and radioactivity determined by liquid scintillation spectrometry (scintillation cocktail Ultima Gold XR; PerkinElmer; counter: LS 6500; Beckman Instruments). The sampling volume removed from the receiver (0.2–0.5 ml) was replaced by fresh solution. Unless stated otherwise, the temperature and RH were 22 °C and 50% RH.

The time courses of ^45^Ca-penetration from CaCl_2_ and Ca-formate were established as described above using ‘Regina’.

The effect of temperature on penetration of ^45^CaCl_2_ was investigated following equilibration of diffusion cells for 1 h at 12, 22 or 35 °C in ‘Regina’. The RH was adjusted to 75%. The receiver was sampled for up to 236 h after droplet application. The number of replications was 10.

The effects of RH on penetration of ^45^CaCl_2_ or ^45^Ca-formate were established in two experiments. In the first experiment, ES excised from ‘Sweetheart’ were mounted in diffusion cells and equilibrated at 0, 28, 75 or 100% RH. The RH was adjusted by holding the diffusion cells above dry silica gel (RH 0%^31^), a saturated slurry of CaCl_2_ (RH 28%^27^) or of NaCl (RH 75%^27^) or above water (RH 100%). Temperature was held constant at 22 °C. Following equilibration, a 5 µl droplet of donor solution was applied. Penetration was monitored by sampling the receiver solution at 0, 1, 5, 20, 48, 80, 120 and 168 h after droplet application. The number of replications was 10. In a second experiment, the effects of increasing RH from 50 to 100% at 96 h after droplet application using ‘Regina’ ES was studied. The increase in RH was achieved by sealing the PMMA holders of the ES on half of the diffusion cells using clear transparent tape (tesa film; tesa SE, Norderstedt, Germany). Because the permeance of the tape to water vapor is markedly lower than that of the ES, the RH above the dried droplet deposit increased rapidly to 100% RH. Un-sealed cells served as controls (RH 50%). Penetration of ^45^CaCl_2_ or ^45^Ca-formate into a receiver solution containing 50 mM CaCl_2_ or Ca-formate, respectively, was monitored by sampling the receiver solutions for up to 216 h. The number of replicates was 10.

The effect of re-wetting the dried-down droplet deposit on the penetration of ^45^CaCl_2_ or ^45^Ca-formate was investigated using ‘Regina’. Diffusion cells were held at 22 °C and RH 50%. The time course of penetration was monitored for up to 20 h as described above. At this time, a 5 µl droplet of deionized water was placed on the dried-down droplet deposit on the ES. Penetration was followed for up to 24 h when the deposit was re-wetted again. A total of five sequential re-wetting cycles was carried out and penetration was monitored for up to 118 h. Diffusion cells without re-wetted deposits served as controls. The number of replications was 10.

### Data analyses

Data are presented as means ± SE. Where error bars are not visible in a graph, they are smaller than the plotted symbols. Data were tested for normality and homogeneity of variance. Thereafter, data were examined using regression analyses, analysis of variance and pairwise t-tests (R version 3.6.3; R Foundation for Statistical Computing, Vienna, Austria) or Tukey’s studentised test (package multcomp 1.3–1, procedure glht, R version 3.6.3; R Foundation for Statistical Computing).

## Data Availability

The datasets generated during the current study are available from the corresponding author on reasonable request.
